# NFAT control of immune function: New Frontiers for an Abiding Trooper

**DOI:** 10.12688/f1000research.13426.1

**Published:** 2018-03-02

**Authors:** Martin Vaeth, Stefan Feske

**Affiliations:** 1 Department of Pathology, New York University School of Medicine, New York, NY, 10016, USA

**Keywords:** NFAT, CRAC, Calcium, STIM, T cell, metabolism, anergy, exhaustion

## Abstract

Nuclear factor of activated T cells (NFAT) was first described almost three decades ago as a Ca ^2+^/calcineurin-regulated transcription factor in T cells. Since then, a large body of research uncovered the regulation and physiological function of different NFAT homologues in the immune system and many other tissues. In this review, we will discuss novel roles of NFAT in T cells, focusing mainly on its function in humoral immune responses, immunological tolerance, and the regulation of immune metabolism.

## Introduction: the NFAT transcription factor family

Nuclear factor of activated T cells (NFAT) was first identified in the late 1980s as part of an inducible nuclear protein complex at the interleukin-2 (IL-2) promoter in activated T cells ^1, 2^. It turned out that this nuclear complex was composed of AP-1 (formed by the transcription factors c-Jun and c-Fos) and a novel family of preformed, cytosolic transcription factors that translocate to the nucleus upon cell stimulation ^3, 4^. NFAT1 (also known as NFATc2 or NFATp), the founding member of this family discovered in 1993 ^3^, and NFAT2 (NFATc1 or NFATc) ^4^ are regulated by the phosphatase calcineurin, which dephosphorylates NFAT factors on an N-terminal regulatory domain and allows them to translocate to the nucleus ^5– 7^. The calcineurin inhibitors cyclosporine A (CsA) and FK506 prevent this dephosphorylation and NFAT nuclear accumulation ^5– 13^. Besides the original NFAT1 and NFAT2, the NFAT family comprises NFAT3 (NFATc4 or NFATx) and NFAT4 (NFATc3), which are also regulated by Ca ^2+^/calcineurin signaling, and the more distantly related NFAT5 (TonEBP) that is predominantly activated by osmotic stress (reviewed in 11, 14– 16). All NFAT proteins share a conserved core region composed of a DNA-binding REL-homology domain and a less conserved N-terminal regulatory domain (also known as NFAT-homology domain). In addition, alternative splicing and the usage of different promoters and polyadenylation sites result in several isoforms that differ in their N and C termini and thus their functional properties. Distinct NFAT family members and their isoforms have both redundant and specific (or even opposing) roles in lymphocyte activation, cell cycle, apoptosis, and cytokine expression, as discussed further below ^11, 14, 17– 20^.

## Activation of NFAT in lymphocytes

**Figure 1.**
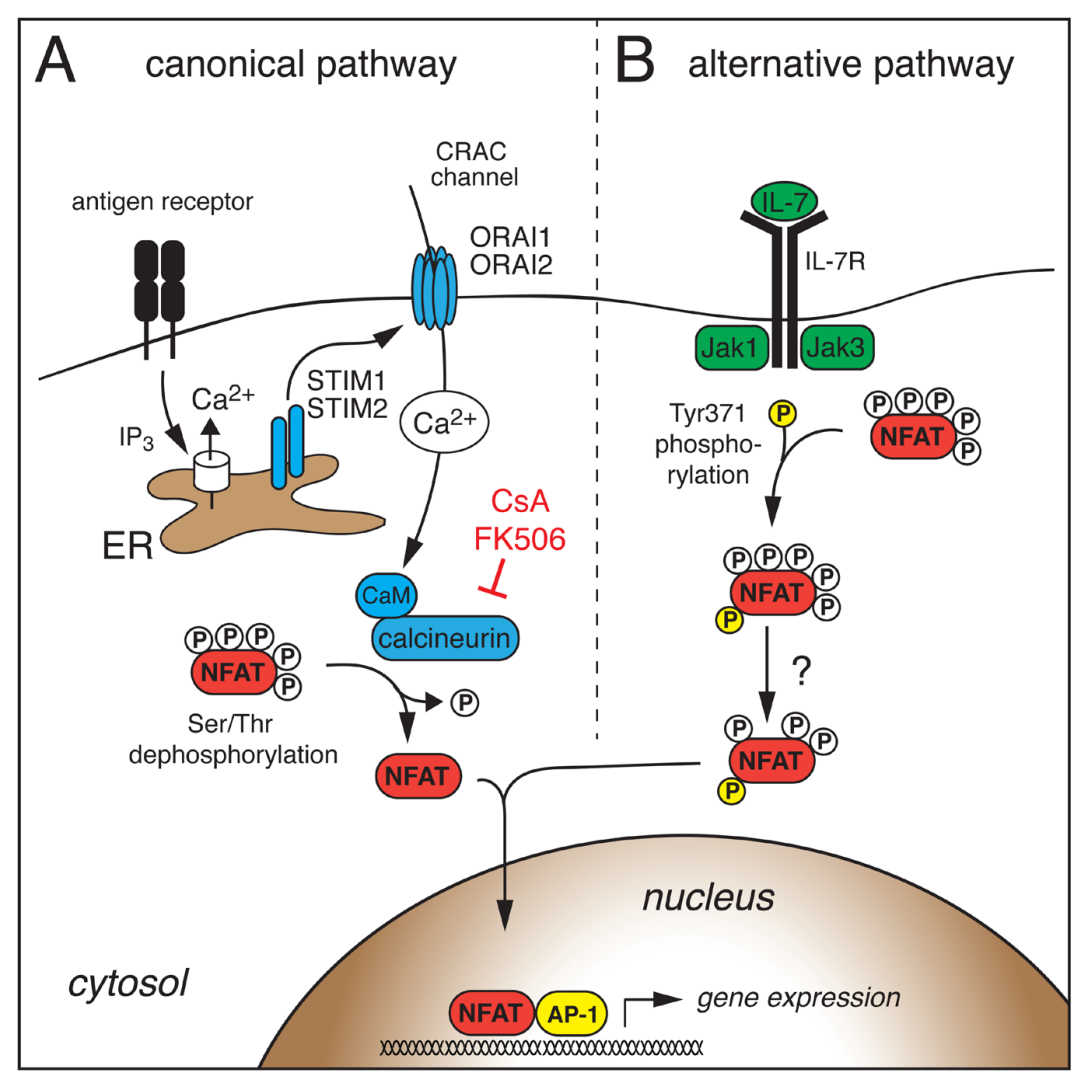
Canonical and alternative NFAT activation in T cells ( **A**) Antigen receptor stimulation leads to the production of inositol-1,4,5-trisphosphate (IP _3_), which opens IP _3_ receptor channels in the endoplasmic reticulum (ER). The subsequent decrease in the ER Ca ^2+^ concentration activates stromal interaction molecule 1 (STIM1) and STIM2, which then bind to and open Ca ^2+^ release-activated Ca ^2+^ (CRAC) channels formed by ORAI1 and ORAI2 proteins in the plasma membrane. Ca ^2+^ influx through CRAC channels activates calmodulin (CaM) and the serine/threonine phosphatase calcineurin. Calcineurin dephosphorylates multiple serine/threonine residues in the regulatory domain of NFAT, resulting in a conformational change, exposure of nuclear localization signals, and nuclear import of NFAT. ( **B**) Janus kinase 3 (Jak3) downstream of the interleukin-7 (IL-7) receptor phosphorylates a single tyrosine residue within the regulatory domain of NFAT2, which induces nuclear translocation and activation of NFAT2 independent of Ca ^2+^ signals and calcineurin in thymocytes. CsA, cyclosporine A; NFAT, nuclear factor of activated T cells.

The canonical NFAT activation pathway by Ca ^2+^/calcineurin signaling has been extensively reviewed ^11, 14, 15, 21^, and we will provide only a brief overview ( Figure 1A). Ligation of T- and B-cell antigen receptors and other receptors that are functionally coupled to phospholipase C (PLC) activation mediates the generation of the second messengers inositol-1,4,5-trisphosphate (IP _3_) and diacylglycerol (DAG). IP _3_ binds to and opens IP _3_ receptor channels in the endoplasmic reticulum (ER), resulting in a decrease in the ER Ca ^2+^ concentration. Dissociation of Ca ^2+^ from ER-luminal EF-hand domains of stromal interaction molecule 1 (STIM1) and STIM2 triggers their translocation to ER–plasma membrane (ER–PM) junctions, where they bind to and activate Ca ^2+^ release-activated Ca ^2+^ (CRAC) channels formed by ORAI1 and ORAI2 proteins in T cells ^22, 23^. The subsequent Ca ^2+^ influx is termed store-operated Ca ^2+^ entry (SOCE), since it is regulated by the Ca ^2+^ concentration in the ER (reviewed in 21, 24). The free cytosolic Ca ^2+^ is bound by calmodulin, which activates the serine/threonine phosphatase calcineurin. Calcineurin dephosphorylates multiple serine residues in the regulatory domain of NFAT, resulting in a conformational change, exposure of nuclear localization signals, and translocation of NFAT into the nucleus ( Figure 1A) ^15, 25, 26^. Recent data suggest that the calcineurin and NFAT interaction is controlled by a high-molecular signaling complex that contains scaffolding proteins (for example, HOMER2 and HOMER3), non-coding RNAs (for example, NRON), and kinases (for example, LRRK2), all of which are required for accurate NFAT activation ^18, 27– 29^. Once in the nucleus, NFAT proteins are rapidly rephosphorylated by nuclear kinases (reviewed in detail in 14, 15, 18). NFAT inactivation is a highly coordinated process in which priming and export kinases within the nucleus phosphorylate different serine residues in the NFAT regulatory domain and initiate the export of NFAT into the cytoplasm. There, maintenance kinases fully phosphorylate NFAT and retain it in the cytoplasm. Different NFAT family members are rephosphorylated by distinct export kinases, including GSK3β (NFAT1 and NFAT2) ^30^, CK1 (NFAT1) ^25^, and DYRK1 (NFAT1 and NFAT2) ^31^, which fine-tune the transcriptional activity of different NFAT homologues by controlling their nuclear residence.

While all NFAT homologues (except NFAT5) are activated by Ca ^2+^/calmodulin and calcineurin-mediated dephosphorylation, NFAT1 and NFAT4 were recently shown to require distinct subcellular Ca ^2+^ and IP _3_ signals for their activation. Whereas Ca ^2+^ influx across the PM is sufficient for NFAT1 activation, NFAT4 requires in addition Ca ^2+^ release from the nuclear envelope triggered by the engagement of IP _3_ receptors ^32– 34^. Moreover, different NFAT homologues differ in their inactivation kinetics. NFAT1 was found to be rephosphorylated more slowly on its regulatory domain than NFAT4, resulting in prolonged NFAT1 activation and residence in the nucleus ^33^. In addition to phosphorylation, NFAT activity can be regulated by protein acetylation ^35^, proteolytic cleavage by caspase 3 ^36^, and SUMOylation by the small ubiquitin-like modifier (SUMO) ^37– 39^. In T cells, SUMOylation of the C termini of NFAT1 and NFAT2 promotes nuclear export of NFAT1 ^38^ and dampens NFAT2-mediated IL-2 transactivation by chromatin condensation ^37^, respectively. The finding that individual NFAT proteins have distinct Ca ^2+^ dependencies for their activation ^32– 34^, different inactivation kinetics ^33^ and are regulated individually by post-translational modifications ^35, 37– 39^ suggests that NFAT homologues are selectively activated on the basis of the strength and type of agonist stimulation ^40^ and the cellular context.

**Figure 2.**
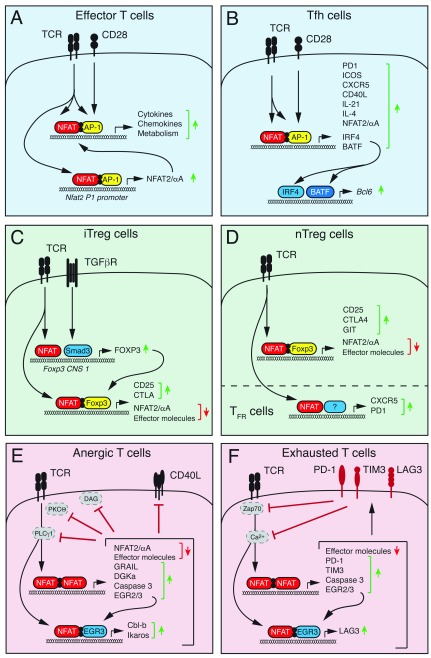
Distinct NFAT functions in effector, follicular, regulatory, and tolerized T cells. ( **A**) Stimulation of conventional T cells via the T-cell receptor (TCR) and co-stimulatory receptors results in NFAT activation and its cooperative DNA binding together with AP-1 (Jun/Fos). NFAT:AP-1 complexes regulate the expression of cytokines and other effector molecules as well as the short NFAT2/αA isoform that further enhances T-cell activation in a positive auto-regulatory loop. ( **B**) In T follicular helper (Tfh) cells, NFAT, together with AP-1, controls the expression of many genes that regulate the differentiation of Tfh cells (IRF4 and BATF), Tfh cell homing to B-cell follicles (CXCR5), and Tfh cell help to B cells (CD40L, IL-4, and IL-21). ( **C**) In peripherally induced regulatory T (iTreg) cells, TCR-dependent NFAT and TGFβ-dependent Smad3 activation converge at the conserved non-coding sequence (CNS) 1 of the *Foxp3* locus to induce Foxp3 expression. ( **D**) In thymus-derived “natural” Treg (nTreg) cells, Foxp3 expression appears largely independent of NFAT activation. In both iTreg and nTreg cells, NFAT forms a ternary NFAT:Foxp3 complex with DNA that induces the expression of Treg-associated genes such as CD25 and CTLA-4 and antagonizes the expression of pro-inflammatory genes and the short NFAT2/αA isoform. In follicular Treg (Tfr) cells, NFAT regulates the expression of CXCR5, CTLA-4, and PD-1 that are required for Tfr cell function. ( **E, F**) Chronic TCR stimulation without co-stimulation triggers the formation of NFAT homomeric complexes that induce a gene expression program associated with T-cell anergy ( **E**) or exhaustion ( **F**) and that is distinct from NFAT:AP-1 complex-mediated gene expression. ( **E**) NFAT-dependent genes associated with anergic CD4 ^+^ T cells include E3 ubiquitin ligases (GRAIL, Itch, and Cbl-b) and caspase 3 that target molecules involved in proximal TCR signaling, which renders T cells unresponsive to re-stimulation. ( **F**) NFAT-dependent genes associated with exhausted CD8 ^+^ T cells are similar to those in anergic CD4 ^+^ T cells but also include inhibitory receptors such as PD-1, TIM3, and LAG3 that antagonize TCR signaling. NFAT, nuclear factor of activated T cells; TGFβ, transforming growth factor beta.

More recently, an alternative NFAT activation pathway independent of Ca ^2+^/calcineurin signaling has been described ( Figure 1B). The common γ (γ _c_) chain cytokine IL-7 can trigger NFAT2 nuclear translocation in double-negative (DN) thymocytes which lack pre-T cell receptor (pre-TCR) signals ^41– 43^. Cytokine-mediated NFAT nuclear translocation was insensitive to CsA, suggesting that the underlying NFAT activation is fundamentally different from the canonical Ca ^2+^/calcineurin signaling pathway. In fact, Janus kinase 3 (Jak3) downstream of the IL-7 receptor directly phosphorylates a single tyrosine residue within the regulatory domain of NFAT2 that induces its nuclear translocation and NFAT-dependent Bcl-2 expression in DN thymocytes ^42^ ( Figure 1B). It remains to be elucidated whether NFAT family members besides NFAT2 are regulated in a similar fashion and whether other γ _c_ cytokines (for example, IL-15 in memory T cells) have similar effects on NFAT activation. Besides post-translational regulation of NFAT activation described above, NFAT activity is further subject to transcriptional regulation of its own expression. The *Nfat2* gene locus encodes six different NFAT2 isoforms (reviewed in 44), including a short NFAT2 isoform (NFAT2/αA) that lacks the C-terminal domain typical of other NFAT proteins ^44– 49^. The expression of NFAT2/αA occurs within hours after TCR stimulation and is dependent on NFAT binding to the isoform-specific P1 promoter ( Figure 2A) ^44^. NFAT2/αA then acts in a positive auto-regulatory loop to induce NFAT-dependent gene expression and T-cell activation. Intriguingly, NFAT2/αA induction occurs in different types of effector T cells, but not in immunosuppressive regulatory T (Treg) cells and exhausted or anergic T cells ^44, 45, 48, 50^, consistent with the idea that high levels of NFAT2/αA favor T helper (Th) cell differentiation and function ( Figure 2A).

## Novel roles of NFAT in humoral immunity

An important role of NFAT in modulating immune responses is due to its transcriptional regulation of numerous cytokines, chemokines, and growth factors in immune cells ^20, 51^. In particular, NFAT is critical for the function and differentiation of Th cells such as Th1, Th2, and Th17 cells (reviewed in 14, 18, 20). Here, we will focus on more recent findings regarding the role of NFAT in shaping humoral immunity and immune tolerance.

**Figure 3.**
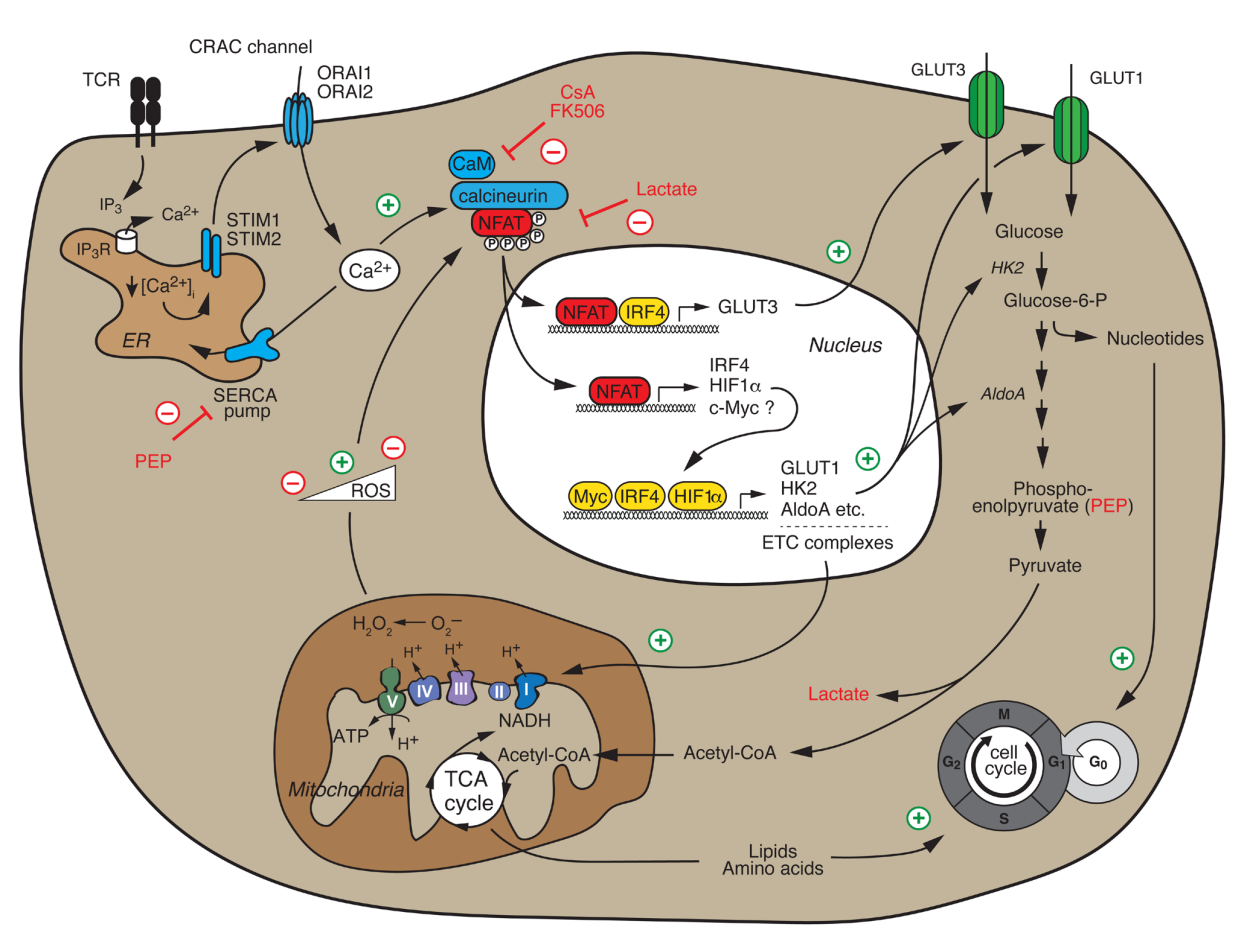
NFAT regulates T-cell metabolism. Ca ^2+^/calcineurin/NFAT signaling following T-cell receptor (TCR) and co-stimulation is required for the switch from catabolic to high-rate anabolic metabolism of activated T cells, cell cycle entry, and proliferation. NFAT directly controls the expression of “metabolic master regulators” such as IRF4, HIF1α, and potentially c-Myc that subsequently induce the expression of glucose transporter 1 (GLUT1), glycolytic enzymes, and mitochondrial electron transport chain (ETC) complexes that mediate aerobic glycolysis and mitochondrial respiration, respectively. In addition, NFAT directly controls the expression of the high-affinity glucose transporter GLUT3. NFAT also senses the metabolic state of T cells. The glycolytic intermediate phosphoenolpyruvate (PEP) inhibits the sarco/endoplasmic reticulum Ca ^2+^ ATPase (SERCA) and thereby enhances Ca ^2+^ signaling and NFAT activation, whereas lactate inhibits NFAT activation. Low levels of reactive oxygen species (ROS) generated by mitochondrial respiration enhance NFAT activation, whereas high ROS levels inhibit NFAT. CRAC, Ca ^2+^ release-activated Ca ^2+^; CsA, cyclosporine A; ER, endoplasmic reticulum; IP _3_, inositol-1,4,5-tisphosphate; NFAT, nuclear factor of activated T cells; STIM, stromal interaction molecule.

Humoral immune responses that result in the production of high-affinity antibodies and the generation of plasma and memory B cells are tightly regulated in the germinal center (GC) reaction. Upon antigen encounter, CD4 ^+^ T follicular helper (Tfh) cells upregulate the chemokine receptor CXCR5 and migrate into B-cell follicles to provide cognate help to GC B cells, thus promoting clonal selection and affinity maturation ^52, 53^. Both Tfh cells and activated (GC) B cells express high levels of NFAT2 (in particular, the short NFAT2/αA isoform) and NFAT1, suggesting a vital role in humoral immunity ^54, 55^. Intriguingly, although NFAT2 regulates activation, antigen presentation, proliferation, and apoptosis of B cells after antigen receptor stimulation *in vitro*, the production of IgG antibodies was largely intact in mice with B cell-specific deletion of NFAT2 ^55, 56^, suggesting that NFAT2 in B cells is not required for the GC reaction and B-cell maturation. Only IgG3 production after immunization with T-cell-independent antigens was modestly impaired, but humoral immune responses following immunization with T-cell-dependent antigens were unaffected, indicating that T-cell-derived signals (for example, CD40L-induced nuclear factor-kappa B [NF-κB] activation) can compensate for the loss of NFAT2 in GC B cells ^57^. These findings are in line with the observation that mice with combined deletion of *Stim1* and *Stim2* genes in B cells and thus abolished activation of all Ca ^2+^-dependent NFAT homologues also showed normal humoral immune responses ^58^. In sharp contrast, mice with T-cell-specific deletion of *Stim1* and *Stim2* ^59^ or *Nfat1* and *Nfat2* ^60^ had strongly impaired GC formation and antibody production after antigen immunization or viral infection, demonstrating that Ca ^2+^/NFAT signaling in T cells, more so than B cells, is required for humoral immunity. At the transcriptional level, NFAT2, together with NFAT1, controls the expression of cell surface receptors, including ICOS, PD-1, CXCR5, and CD40L, and cytokines such as IL-4 and IL-21 that are essential for Tfh cell differentiation, GC formation, and B-cell affinity maturation ^54, 59– 64^. Furthermore, NFAT2 controls the expression of the “pioneering” transcription factors IRF4 and BATF in Tfh cells ^59, 65^, and both factors, though not specific for Tfh cells, are required for Bcl-6 expression and thus Tfh cell lineage commitment ^52^ ( Figure 2B). Since IRF4 was shown to regulate glycolysis and mitochondrial respiration in T cells ^66, 67^, NFAT may control Tfh cell differentiation at least in part through IRF4 expression and metabolic reprogramming of CD4 ^+^ T cells ^68^ ( Figure 3) (see below).

## NFAT in peripheral immune tolerance

NFAT controls the expression of many pro-inflammatory cytokines such as interferon gamma (IFNγ), IL-4, and IL-17 in Th1, Th2, and Th17 cells, respectively ^14, 20^, making it and its upstream regulators prime molecular targets for the suppression of auto- and allo-immune responses. On the other hand, NFAT also has important roles in immune tolerance. NFAT controls the differentiation and function of Treg cells ^54, 69– 74^ and IL-10-producing regulatory B (Breg) cells ^55, 58, 75^ that are required for immune homeostasis and crucial to prevent auto-immunity ( Figure 2C,D). Treg cells are characterized by the expression of the transcription factor Foxp3, which is critical for their function ^76^. Two main groups of Foxp3 ^+^ Treg cells exist: natural Treg (nTreg) cells that develop in the thymus and represent a professional and stable T-cell lineage and induced Treg (iTreg) cells that differentiate from naïve CD4 ^+^ T cells in the periphery after antigen receptor stimulation in the presence of transforming growth factor beta (TGFβ) and that are short-lived ^76, 77^. Foxp3 expression is regulated differently in nTreg and iTreg cells through distinct regulatory conserved non-coding sequences (CNSs) in the *Foxp3* gene locus ^78^. In iTreg cells, NFAT binds to CNS1 together with Smad3 and facilitates TCR- and TGFβ-induced Foxp3 expression ^72, 73, 78^, whereas CNS1 is dispensable for nTreg cell development ^78^ ( Figure 2C). In differentiated nTreg cells, NFAT binds to CNS2 that spatially interacts with its promoter to maintain stable Foxp3 expression ^70, 78^. Because of the use of different CNS elements, Foxp3 expression in iTreg and nTreg cells depends, to varying degrees, on NFAT signaling ^73, 79^. Foxp3 expression is cooperatively regulated by NFAT1, NFAT2, and NFAT4 in iTreg cells, and the deletion of just one NFAT family member significantly reduced Foxp3 expression ^73^. By contrast, ablation of NFAT1, NFAT2, or NFAT4 or combined deletion of two NFAT genes did not perturb Foxp3 expression in nTreg cells ^73, 80, 81^. In addition, the suppressive function of NFAT-deficient Treg cells *in vitro* and *in vivo* was largely preserved in the absence of NFAT1, NFAT2, or NFAT4 ^73, 80– 82^.

In Treg cells, NFAT1 was shown to form a ternary complex with Foxp3 at the *Il2* promoter that replaces AP-1 (Jun/Fos) in the NFAT:AP-1 complex present in effector T cells ^69, 74^. Mutations that disrupt these NFAT:Foxp3 complexes eliminate the suppressive function of Treg cells ^69, 74, 83^. Foxp3 thus transforms a transcriptionally activating NFAT:AP-1 complex into a repressive NFAT:Foxp3 complex ^74, 83^ ( Figure 2C,D). Since many NFAT:AP-1-regulated genes, such as cytokine or chemokine genes, are pro-inflammatory and are not highly expressed in Treg cells, it is plausible that NFAT:Foxp3 complexes function as a brake for the transcriptional activity of NFAT ^71, 83^. Likewise, inducible cAMP early repressor (ICER), a dominant-negative splice form of cAMP-responsive element modulator (CREM) that is highly expressed in Treg cells ^84^, may replace AP-1 and form heteromeric repressive complexes with NFAT in Treg cells ^85, 86^. In addition to “neutralizing” the transcriptional activity of NFAT, Foxp3 directly suppresses the expression of NFAT2/αA ^45, 73, 79, 87^, which is strongly induced in activated effector T cells ^44, 45, 50^, thereby limiting the amount of transcriptionally active NFAT in Treg cells. It is noteworthy that despite its reduced expression and activity in Treg cells, NFAT fulfills some crucial functions in specific Treg cell subsets. For instance, NFAT2 controls the induction of CXCR5 in T follicular regulatory (Tfr) cells and thus facilitates their homing to GCs ^54, 59^, where they limit the GC reaction to prevent humoral auto-immunity ^54, 88, 89^. Likewise, NFAT controls the expression of the inhibitory co-receptor CTLA-4 on Tfr cells ^69, 74^ that is important to limit the GC reaction ^89^ ( Figure 2D). It remains to be elucidated whether NFAT in general or individual homologues play important roles in other specialized Treg cell subsets (such as tissue-resident Treg cells) that have intriguing features beyond immune regulation ^90^.

## NFAT controls T-cell anergy and exhaustion

Suboptimal or chronic stimulation in the absence of adequate co-stimulation induces clonal anergy and exhaustion in CD4 ^+^ and CD8 ^+^ T cells, respectively ^91– 96^. In both cases, T cells become hyporesponsive (that is, tolerant), thus preventing damage by auto-reactive or persistently activated T cells. In CD4 ^+^ T cells, antigen stimulation without appropriate co-stimulatory signals induces NFAT activation but—owing to the absence of MAPK signaling—without the formation of canonical NFAT:AP-1 complexes. Instead, NFAT forms homodimers or complexes with other transcription factors, such as EGR2 and EGR3, that activate a distinct, tolerogenic gene expression program ^97– 99^. Among these “anergy-inducing genes” are E3 ubiquitin ligases (such as Cbl-b, Itch, and Grail), diacylglycerol kinase α (DGKα), and caspase 3 that promote the induction of T-cell anergy ^91, 92, 98, 100^. Itch, Cbl-b, and caspase 3 target proximal TCR signaling molecules such as PLCγ 1 and PKCΘ for degradation, whereas DGKα and GRAIL interfere with co-stimulatory pathways and CD40L signaling ^91, 92, 98, 100^, resulting in clonal unresponsiveness to re-stimulation ( Figure 2E). The contribution of individual NFAT family members (and their isoforms) to anergy induction is not fully understood, but the phenotypes of different *Nfat*-deficient mice suggest that NFAT1 and NFAT4 are involved in anergy induction ^98, 101– 106^ but that NFAT2, especially NFAT2/αA, has the opposite function and may contribute to the reversal of anergy ^46^.

Similar to CD4 ^+^ T-cell anergy, chronic antigen stimulation (for example, in the context of cancer or chronic infection) promotes a gradual loss of effector functions in T cells, known as T-cell exhaustion ^94, 95^. Though mainly studied in CD8 ^+^ T cells, exhaustion has also been reported in CD4 ^+^ T cells, where it was shown to depend on the activity of NFAT1 ^106^. Like anergy, exhaustion is induced by impaired NFAT:AP-1 cooperation, but the underlying transcriptional program leading to unresponsiveness appears to be slightly different from anergy ^91– 93^. Martinez *et al*. ^93^ demonstrated a critical role for NFAT in T-cell exhaustion by using an engineered, constitutively active mutant of NFAT1 that is unable to form cooperative complexes with AP-1 (CA-NFAT1-RIT, named after mutations in the R468, I469, and T535 residues that mediate the interaction of NFAT with AP-1) ^93, 98, 99^. Ectopic expression of CA-NFAT1-RIT in CD8 ^+^ T cells impaired effector functions and induced a gene expression profile that was highly reminiscent of exhausted and anergic T cells ^93^. T cells expressing CA-NFAT1-RIT showed defective signaling and Ca ^2+^ mobilization after TCR crosslinking that correlated with the upregulation of E3 ligases and caspase 3, which may induce the degradation of signaling molecules similar to anergic T cells ^91– 93^. In addition, CA-NFAT1-RIT binds directly to regulatory regions of exhaustion-associated genes in CD8 ^+^ T cells, including the inhibitory receptors PD-1 and TIM3, and induces their expression ^61, 93^ ( Figure 2F). Collectively, these data suggest that NFAT forms homomeric or cooperative complexes with activating or repressive transcription factors that determine the phenotype and function of T cells.

## NFAT regulates T-cell metabolism

Emerging evidence shows that NFAT—in particular, NFAT2—acts as a central regulator of T-cell metabolism ( Figure 3) ^68, 107–110^. Naïve T cells are metabolically quiescent and characterized by minimal nutrient uptake, low glycolysis, and effective oxidative phosphorylation ^111– 113^. By contrast, activated lymphocytes reset their metabolism to a high-rate anabolic metabolism fueled by aerobic glycolysis that supports the synthesis of macromolecules required for clonal expansion ^111, 112, 114^. We recently showed that CD4 ^+^ T cells lacking *Stim1* and *Stim2*, and thus SOCE and NFAT activation, failed to undergo this “glycolytic switch” and antigen receptor-induced clonal expansion ^68^. SOCE is required for the expression of glucose transporters GLUT1 and GLUT3 as well as numerous glycolytic enzymes, including hexokinase 2, phosphoglycerate kinase, and aldolase A that metabolize glucose and produce anabolic intermediates required for clonal expansion ^68^. The majority of SOCE-dependent glycolytic genes are dependent on NFAT-mediated transcription, as evident from their impaired expression in T cells of *Nfat1/Nfat2*-deficient mice and increased expression in T cells expressing a constitutively active form of NFAT2 ^68, 110^. In agreement with our data, Klein-Hessling *et al*. recently showed that NFAT2 controls metabolic gene expression, the glycolytic switch, and thus the function of cytotoxic CD8 ^+^ T cells ^110^. Although NFAT2 is essential for glycolytic gene expression, most glycolysis genes (with the exception of GLUT3 and HK2) do not show robust NFAT2 binding in chromatin immunoprecipitation assays, suggesting an indirect regulation by NFAT ^68, 110^. Instead, SOCE and NFAT2 control the expression of transcriptional regulators of glycolysis such as IRF4 ^59, 68^, HIF-1α ^68, 115^, and, by some accounts, c-Myc ^68, 107, 110, 116– 118^. Overexpression of NFAT2 in SOCE-deficient T cells rescued the expression of IRF4 and GLUT1 and partially restored the proliferation of T cells ^68^. It is noteworthy that alternative activation of NFAT via the γ _c_ cytokines IL-2 and IL-7 does not require SOCE to induce glycolytic gene expression and proliferation of T cells ^68^. Moreover, the addition of exogenous IL-2 to NFAT2-deficient CD8 ^+ ^T cells restores their defective glycolysis ^110^. Besides glycolysis, SOCE and calcineurin also regulate mechanistic target of rapamycin (mTOR) signaling, oxidative phosphorylation, and mitochondrial gene expression ^68, 107^, suggesting that the Ca ^2+^/calcineurin/NFAT pathway plays important and yet-to-be-defined roles in controlling cell metabolism. These data also provide a compelling new mechanism to explain the potent immunosuppressive effects of the calcineurin inhibitors tacrolimus and CsA by interfering with lymphocyte metabolism.

NFAT not only instructs T-cell metabolism but also senses the metabolic state of T cells and nutrient availability. Glucose-deprived T cells had impaired nuclear translocation of NFAT1 (but not NFAT2), which correlated with reduced IFNγ and CD40L expression and anti-tumor immunity ^108^. The glycolytic intermediate phosphoenolpyruvate (PEP) acts as a “metabolic checkpoint” that supports the rapid nuclear translocation of NFAT1. PEP was shown to inhibit the sarco/endoplasmic reticulum Ca ^2+^ ATPase (SERCA), which pumps Ca ^2+^ from the cytosol into the ER ^108^, resulting in increased intracellular Ca ^2+^ levels and NFAT1 activation. Furthermore, reactive oxygen species (ROS) generated by mitochondrial respiration were shown to modulate the nuclear translocation of NFAT1 in a concentration-dependent manner ^107, 109^. TCR-induced low (physiological) levels of mitochondrial ROS promote nuclear translocation of NFAT1 ^109^. Higher ROS levels inhibit NFAT activation ^107^, suggesting that redox regulation fine-tunes NFAT function, although the underlying mechanisms remain unknown. In addition, high extracellular lactate levels (as encountered, for instance, in the tumor environment) were shown to result in intracellular acidification of CD8 ^+^ T cells and thus inhibition of NFAT2 induction, IFNγ expression, and anti-tumor immunity ^119^. These findings suggest that NFAT not only regulates T cell metabolism but, in addition, may sense nutrient availability and the bioenergetic status of T cells.

## Concluding remarks and future directions

Different T-cell subsets use distinct metabolic programs ^113, 120^. Whereas CD4 ^+^ effector Th1, Th2, Th17, and CD8 ^+^ cytotoxic T cells are thought to depend largely on glycolysis and glutaminolysis, memory, follicular, and Treg cells preferentially use mitochondrial respiration and lipid oxidation ^113, 114, 120^. In addition, exhausted and anergic T cells are bioenergetically distinct from their functional counterparts. The SOCE/calcineurin/NFAT pathway emerges to play important roles in the regulation of T-cell metabolism, but the details of this regulation remain to be fully understood. Moreover, individual NFAT family members and even their isoforms can have opposite roles in T-cell proliferation, anergy, and/or exhaustion that may extend to metabolism. Ectopic expression of NFAT2, for instance, promotes cell cycle progression and proliferation *in vitro*, whereas NFAT1 expression inhibits proliferation and induces apoptosis ^121^. Similar observations were made in different NFAT-deficient mice. Whereas the deletion of *Nfat2* impairs TCR-induced proliferation ^82, 110, 122, 123^, T cells from *Nfat1 ^–/–^* and *Nfat1 ^–/–^Nfat4 ^–/–^* mice are, however, hyperproliferative in response to various stimuli ^102, 104, 105^. Future work will have to unravel how individual NFAT family members and their isoforms in combination with distinct transcriptional partners regulate the function of effector and Treg cells and the induction of exhaustion/anergy in the context of tumors, auto-immunity, and infection.
